# Automatic Emotion Perception Using Eye Movement Information for E-Healthcare Systems

**DOI:** 10.3390/s18092826

**Published:** 2018-08-27

**Authors:** Yang Wang, Zhao Lv, Yongjun Zheng

**Affiliations:** 1 School of Computer Science and Technology, Anhui University, Hefei 230601, China; e16201094@stu.ahu.edu.cn; 2 Institute of Physical Science and Information Technology, Anhui University, Hefei 230601, China; 3 School of Electronics, Computing and Mathematics, University of Derby, Derby DE22 3AW, UK; y.zheng@derby.ac.uk

**Keywords:** emotion recognition, EOG, eye movement video, healthcare, adolescence

## Abstract

Facing the adolescents and detecting their emotional state is vital for promoting rehabilitation therapy within an E-Healthcare system. Focusing on a novel approach for a sensor-based E-Healthcare system, we propose an eye movement information-based emotion perception algorithm by collecting and analyzing electrooculography (EOG) signals and eye movement video synchronously. Specifically, we extract the time-frequency eye movement features by firstly applying the short-time Fourier transform (STFT) to raw multi-channel EOG signals. Subsequently, in order to integrate time domain eye movement features (i.e., saccade duration, fixation duration, and pupil diameter), we investigate two feature fusion strategies: feature level fusion (FLF) and decision level fusion (DLF). Recognition experiments have been also performed according to three emotional states: positive, neutral, and negative. The average accuracies are 88.64% (the FLF method) and 88.35% (the DLF with maximal rule method), respectively. Experimental results reveal that eye movement information can effectively reflect the emotional state of the adolescences, which provides a promising tool to improve the performance of the E-Healthcare system.

## 1. Introduction

E-Healthcare systems, an effective and timely communication mode between patients, doctors, nurses, and other healthcare professionals, has been a research hotspot in the field of intelligent perception and healthcare for several years [1,2,3]. Traditional healthcare systems mainly depend on a large number of paper records and prescriptions, making them old-fashioned, inefficient, and unreliable [4]. With the rapid development of computer technology, the implementation of sensor-based E-Healthcare systems has attracted increasing attention. So far, the existing E-Healthcare systems focus mainly on the acquisition and recording of information associated with physical health conditions (e.g., body temperature, saturation of pulse oxygen, respiratory rate, heart rate, etc.), while ignoring the emotional health aspect. As a matter of fact, emotional health plays a very important role in improving the effectiveness of a patient’s rehabilitation therapy, which has become an important factor in designing E-Healthcare systems [5,6,7].

In recent years, researchers have carried out a series of studies on the automatic acquisition and analysis of emotional states [8,9,10,11]. Currently, the commonly used emotional information acquisition methods are mainly divided into contact-free and contact two ways. Among them, contact-free methods mainly refer to speech, facial expressions, postures, etc. Such methods have the advantages of simple signal acquisition and causing no discomfort to the subjects. However, when the subjects intentionally mask their emotions, the actual emotional state is not consistent with the external performance. In such a case, it is difficult for contact-free methods to make correct judgements. By contrast, the contact methods can effectively overcome the abovementioned problem due to the undeceptiveness of physiological signals. Generally, peripheral physiological signals acquired from contact bioelectrodes mainly include electroencephalograms (EEG), electrooculograms (EOG), electromyography (EMG), body temperature, etc. Among them, the EEG-based emotion recognition has attracted widespread attention. For instance, Zhang et al. [12] classified users’ emotional responses into two states with an accuracy of 73.0% ± 0.33% during image viewing. Kwon et al. [13] combined EEG and galvanic skin response (GSR) signals and obtained an emotional classification accuracy of 73.4% by using a convolution neural networks model. Lin et al. [14] explored emotion-specific EEG features to classify four music-induced emotional states and obtained an average classification accuracy of 82.29% ± 3.06%. 

Considering the following three facts: (1) as the main perception approach to acquire the information of external scenes, users will roll their eyeballs to search for information of interest [15]. Therefore, a very close relationship may exist between the eye movement activities (e.g., saccades, fixations, blinks, etc.) and the scene content. That is, the eye movement patterns presented by the user may reflect the emotional states induced by the scene information. (2) The EOG method provides another possibility to record eye movement other than the video method [16]. Because of the advantages of rich emotional information, low cost, light computational burden, less influence of environmental illumination, etc., the EOG method has the potential to develop a long-term wearable emotion monitoring system. (3) Adolescence is a key period in the development of psychopathological symptoms up to full-blown mental disorders [17,18]. During this period, individuals are prone to a large number of psychological problems and many mental illnesses make their first appearance [19,20,21]. Therefore, using eye movement information to perceive emotional states plays an important role of adolescents’ healthcare, which can provide an effective supplement to EEG-based emotion recognition.

The existing works about eye movement information processing mainly focus on the analysis of the relationships between eye movement signals and cognitive requirement. Among them, Partala [22] and Bradley [23] et al. used music and pictures respectively, to induce emotions, and observed the changes in subjects’ pupils. They found that the pupil diameter induced by positive or negative stimuli was significantly larger than under neutral conditions. In addition, these changes in the pupils and the electrical responses of the skin presented a high similarity due to the regulation of the sympathetic nervous system. Shlomit et al. [24] explored the relevance induced gamma-band EEG and saccade signals during different cognitive tasks. Their experimental results revealed that eye movement patterns were associated with cognition activities. Jiguo et al. [25] investigated how processing states alternate in the cases of different mental rotation tasks by acquiring and analyzing eye movement signals. They proved that the procedure of mental rotation consisted of a series of discrete status that were accomplished in a piecemeal fashion.

However, eye movement signals are both varied in the time and frequency domains according to different emotional states. For instance, the speed of rolling eyeballs will increase when the user feels excitement; similarly, it will decrease when the user feels sad. Thus, there is a large limitation in performance improvement based on the time-domain analysis method due to the complexity of the emotional eye movement signals. In this paper, to achieve a detailed representation of emotional eye movement by analyzing the changes of time-domain and frequency-domain features, we propose a time-frequency features extraction method on the combination of video and EOG signals. Specifically, we used audio-video stimuli to elicit three types of emotions on the valence dimension, e.g., positive, neutral, and negative. On the basis, we collected EOG signals and eye movement videos synchronously, and extracted their time/frequency features of different eye movement patterns such as saccade, fixation, pupil diameter, etc. Furthermore, we explored the feature fusion strategies to improve the ability of emotion representation. Finally, the Support Vector Machine (SVM) was adopted as a classifier to identify the emotional states. The paper is organized as follows: Section 2 introduces the preliminaries, and Section 3 describes the proposed eye movement-based emotion recognition method in detail. Our experiments and results analysis are presented in Section 4. Section 5 concludes this work.

## 2. Preliminaries

**Figure 1:**
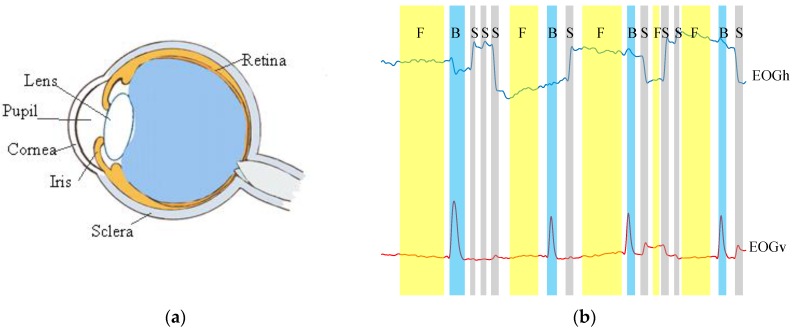
(**a**) The anatomy of eyeball; (**b**) The denoised horizontal ($EOG_{h}$) and vertical ($EOG_{v}$ ) signal components. Three eye movement types are marked in gray: saccades (S), fixations (F), and blinks (B).

The eyeball can be modeled as a dipole, with a positive pole located at the cornea and a negative pole at the retina (shown in Figure 1a). When the eyeball rolls from the center to the periphery, the retina approaches one of the electrodes and the cornea approaches the opposite electrode. This variation in dipole orientation results in a change in the potential field, which can be recorded by EOG. Generally, there are three basic eye movements types that can be detected using the EOG method, i.e., saccades, fixations, and blinks (see Figure 1b). Their definitions are depicted as follows:

• Saccades

When viewing a visual scene, the eyeball constantly moves to create a mental “map” from the interest region of the scene. This perception procedure can be implemented by the fovea which is a small area in the center of the retina. The simultaneous movement of two eyeballs is called a saccade [26]. The average saccade duration is from 10 to 100 ms [27].

• Fixations

Fixation is a static state of the eye movement. In the visual scene, the gaze is fixed in a specific position. Fixations are usually defined as the time between each two saccades. The duration is 100 to 200 ms [28].

• Blinks

Blinking is a rapid eye movement that is caused by the movement of the orbicularis muscle, the frontal part of the cornea is coated with a thin liquid film, the so-called “precornial tear film”. Diffusion of liquids to the surface of the cornea requires frequent opening and closing of the eyelids, or blinking. The average blink duration is between 100 and 400 ms [26].

## 3. Methods

**Figure 2:**
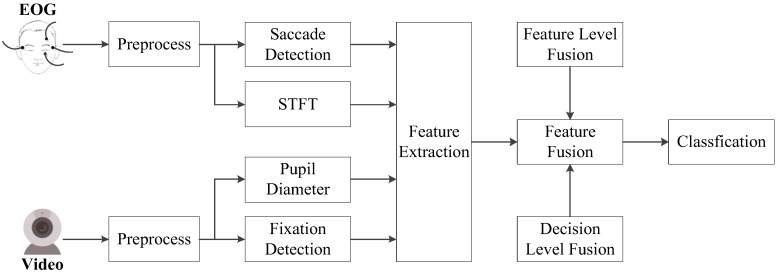
The architecture of the proposed emotion recognition algorithm based on eye movement information.

Figure 2 shows the overall architecture of emotion recognition based on eye movements. In the first step, raw EOG and eye movement video signals are preprocessed to remove artifacts. Next, time-frequency features extracted by the Short-Time Fourier Transform (STFT) [29] as well as time domain features according to saccades, fixations, and pupil diameters are calculated from the processed eye movement data. Finally, two fusion strategies are explored to improve the performance of the proposed emotion recognition algorithm.

### 3.1. Data Acquisition

**Figure 3:**
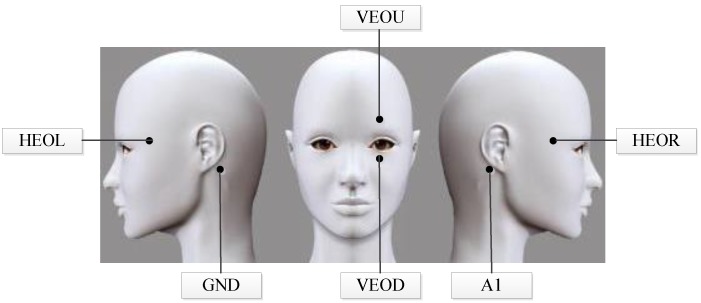
Demonstration of electrodes distribution.

EOG signals are acquired by six Ag-AgCl electrodes together with a Neuroscan system (Compumedics Neuroscan, Charlotte, NC, USA). The distribution of electrodes is shown in Figure 3. Specifically, electrodes HEOR and HEOL fixed on the outer side of each eyeball with 2 cm are used to detect the horizontal EOG signals. Similarly, electrodes VEOU and VEOD arranged 2 cm above and below the left eyeball are applied to collect vertical EOG signals. Reference electrode A1 and ground electrode GND are attached to the left and right mastoid, respectively. Besides, an infrared camera with the resolution of 1280 × 720 is utilized to capture eye movement video signals. The height of the camera is the same level as the subject’s eyeball and the distance between the camera and the subject is 25 cm. In order to collect EOG and video signals synchronously, we use a specific acquisition software developed in our laboratory.

As for emotional stimuli selection, literatures [30] have proven the feasibility of using video clips to induce emotions. Thus, we choose comedy or funny variety video clips to induce positive emotional state, landscape documentary for neutral state, and horror film or tragedy clips for negative state [31,32]. To determine which optimal movie clips can induce the emotion effectively, we perform the movie clips optimization operation according to the following steps. Firstly, we keep the clips as short as possible to avoid multiple emotions. Thus, 60-s highlighted video segments with maximum emotional content from each of original stimuli are manually extracted. Then, we select 120 test clips preliminarily from 180 highlighted videos by a self-assessment software also designed in our laboratory. Furthermore, we scale the emotional intensities into five levels ranged from unpleasant (level 1) to pleasant (level 5). Next, we invite ten volunteers who are not involved in the following emotional recognition experiment to view all stimuli as many times as he/she desired and score them. In order to choose the most representative stimuli clips, we define the normalized score, x, as follows:

(1) $$score(x)=\frac{\mu_{x}}{\sigma_{x}},$$

where $\mu_{x}$ is the mean value and $\sigma_{x}$ indicates the standard deviation. In this way, we can obtain the average score values for each stimulus clip for all volunteers. The higher the normalized score is, the stronger the emotional intensity is. Finally, we determine the highest rated 72 movie clips as stimuli in the emotion recognition experiments.

**Figure 4:**
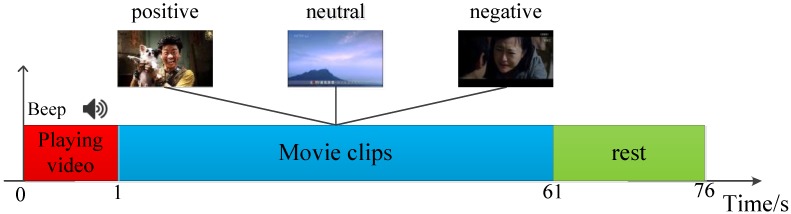
The experimental paradigm of each trial. The horizontal axis indicates the duration of the experiment.

On this basis, we design an experimental paradigm (as shown in Figure 4) to acquire the emotional eye movement signals. Each trial starts with a presentation of a word “begin” displayed on the screen to prompt playing movie clip, followed by a short warning tone “beep”. Afterwards, three different emotion movie clips with the duration of 60 s are displayed at random. When the movie clip ends, the subject is allowed to relax for 15 s in order to attenuate the current induced emotion.

In the present work, eight healthy subjects (five females and three males, aged 24 ± 1) are involved in the experiment. All of them have normal or corrected-to-normal vision. Every subject is required to seat about 40 cm in front of the screen. Stereo loudspeakers are located on the desktop, and the sound volume is empirically initialized to a reasonable level. Before the experiment, all subjects are informed the purpose and the procedure of the experiments and arranged to preview 2–3 stimuli to be familiar with the experimental environment and instrument. Besides, they will be asked whether the conditions such as distance between subject and screen, the volume, the indoor temperature, etc., are comfortable. If not, the setting will be adjusted according to subject’s feedback. 

### 3.2. Data Preprocessing

Generally, the frequency of EOG signals is concentrated in 0.1 to 38 Hz and its main components are within 10 Hz [33]. Considering the following two aspects: (1) maintaining the effective emotional eye movement components, as well as removing based-line drift caused by background signals or electrode polarization; (2) suppressing noises exceed 10 Hz such as high frequency components of different artificial sources, electrodes movement, power line noise, etc. We adopt a 32-order band-pass digital filter with cut-off frequency of 0.01–10 Hz to preprocess the raw EOG signals [34]. In addition, the frame rate of the collected eye movement video is 30 frames per second and the sampling ratio of the EOG signals is 250 Hz.

### 3.3. Features Extraction

#### 3.3.1. Time-Frequency Domain Features Extraction

**Figure 5:**
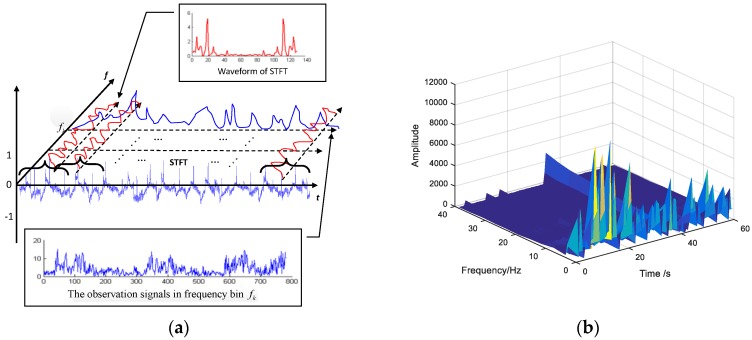
Time-frequency features extraction. (**a**) Demonstration of acquiring the frequency-domain observation vectors; (**b**) Time-frequency diagram after STFT.

Suppose $x_{i}\left(t\right)$ is the preprocessed EOG signals, its L-point STFT can be computed as follows:

(2) $$X_{i}(f,\tau )=\sum_{t=0}^{L-1}x_{i}(t)win(t-\tau )\exp(-j2\pi \frac{f}{f_{s}}t)\;i=1,\cdots ,4;\tau =\tau_{0}\cdots \tau_{M-1},$$

where *win*(*t)* is a Hamming window function, and *τ* indicates the index of sliding windows. *f_s_* represents the sampling ratio, $f_{l}=\frac{f_{s}}{L}l,l=0,1,\cdots L-1$ are discrete frequency points. After STFT, the time-domain observation signal *x_x_*(*t*) is transformed into a new L×M frequency-domain observation matrix:

(3) $$STFT[x_{i}(t)]=\left[\begin{array}{cccc} X_{i}(f_{0},\tau_{0}) & X_{i}(f_{0},\tau_{1}) & \cdots & X_{i}(f_{0},\tau_{M-1})\\ X_{i}(f_{1},\tau_{0}) & X_{i}(f_{1},\tau_{1}) & \cdots & X_{i}(f_{1},\tau_{M-1})\\ \vdots & \vdots & \vdots & \vdots \\ X_{i}(f_{L-1},\tau_{0}) & X_{i}(f_{L-1},\tau_{1}) & \cdots & X_{i}(f_{L-1},\tau_{M-1}) \end{array}\right],$$

where *M* is the total number of windowed signal segments, variable i represents the index of the collection electrodes. The procedure of acquiring frequency-domain observation vectors is demonstrated in Figure 5a.

If we extract all data as features along with time-axis at frequency bin $f_{k}(k=0,1,\cdots ,L-1)$, the time-frequency domain observation vectors can be acquired:

(4) $$X(f_{k})=\left[X_{i}(f_{k})\right]\Rightarrow [X_{i}(f_{k},\tau_{0})\cdots X_{i}(f_{k},\tau_{M-1})]\;k=0,\cdots ,L-1,$$

Clearly, the length of frequency-domain observation vectors is equal to the number of sliding windows. The time-frequency diagram of an EOG segment after STFT is shown in the Figure 5b.

#### 3.3.2. Time Domain Features Extraction

Lu et al. [30] has proved that there is no significant difference in the blink duration among different emotional states. Therefore, we extract the time-domain features of saccade, fixation, and pupil diameter from each eye movement signal segment. The details are listed in Table 1.

• Saccade duration detection

**Figure 6:**
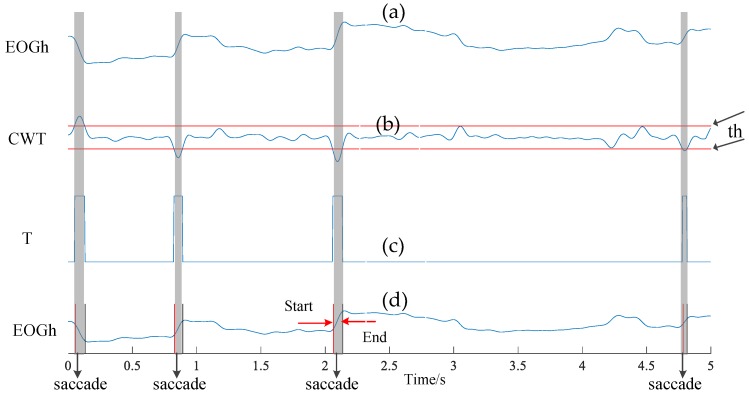
The process of saccade detection. (**a**) Raw horizontal EOG signal; (**b**) wavelet coefficient with detection threshold ±th; (**c**) the detection results of saccade and non-saccade, and (**d**) corresponding saccade segments in terms of outputs from subfigure (**c**).

Detecting the saccade segments is an important step to acquire saccade time. Here, we adopt the Continuous Wavelet Transform (CWT) algorithm [26] to detect saccades. Suppose $x_{h}(t)$ is the preprocessed horizontal EOG signal components, the CWT procedure can be expressed as:

(5) $$C_{b}^{a}(s)=\int_{IR}x_{h}(t)\frac{1}{\sqrt{a}}\psi \bar{\left(\frac{t-b}{a}\right)}dt,$$

where ψ is the mother wavelet, $C_{a}^{b}$ indicates the wavelet coefficient at scale a and position b. In our work, we adopt the Haar function as the mother wavelet, and empirically initialize the scale with 20. Furthermore, we employ a pre-defined threshold $th_{sac}$ on the wavelet coefficient C (demonstrated with red lines in Figure 6b). If the absolute value of $th_{sac}$ is higher than C, the detection result *T* is set to 1; otherwise, *T* is 0. This procedure can be depicted as follows:

(6) $$T=\{\begin{array}{cc} 1 & C\leq -th_{sac},C\geq th_{sac}\\ 0 & -th_{sac}<C<th_{sac} \end{array},$$

In this way, we can transform the wavelet coefficient C to a series of rectangular pulses as shown in Figure 6c. We record the rising edge of the rectangular pulse as the start-point of saccade, and the falling edge as the end-point (see Figure 6d). Additionally, in order to improve the accuracy, we remove the detected result exceeding 10–100 ms in terms of the scope of saccade duration [27].

• Pupil diameter

Initially, we calculate the weighted average of R, G, B components of each frame acquired from the raw facial video to obtain the grayscale image of each frame. Then, we use a detection threshold *th_bi_* to obtain the binary graph which can represent the approximate contour of the pupil. That is, if the value of the current pixel is higher than this threshold *th_bi_*, the current pixel is set to 1 (white); otherwise it set to 0 (black). To obtain the radius and center of the pupil, we further employ the Hough transform [35] to process the binary image. The basic idea of the Hough transform for pupil size detection is to map the edge pixel points in the image space to the parameter space, then accumulate the accumulated values corresponding to the coordinate point elements in the parameter space, and finally determine the center and radius according to the accumulated value.

The circular general equation is ${\left(x-a\right)}^{2}+{\left(y-b\right)}^{2}=r^{2}$, here, (a,b) is the center of the circle and r is the radius. In a rectangular coordinate system, the point (x,y) on the circle can be converted to the polar coordinate plane. The corresponding equation is expressed as:

(7) $$\left\{ \begin{array}{c} x=a+r\cos\theta \\ y=b+r\sin\theta \end{array}\;\theta =[0,2\pi ),\right.$$

An edge point $\left(x_{0},y_{0}\right)$ in the image space is mapped to the parameter space with radius $r_{0}$. Substitute this edge point into Equation (6) and then perform the following transformation:

(8) $$\left\{ \begin{array}{c} a=x_{0}-r_{0}\cos\theta \\ b=y_{0}-r_{0}\sin\theta \end{array},\right.$$

**Figure 7:**
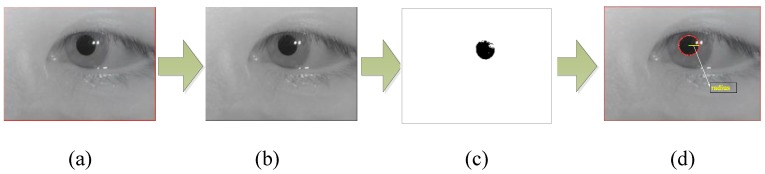
The process of extracting the pupil diameter. (**a**) The original eye movement frame picture; (**b**) the grayscale image; (**c**) the binary image, and (**d**) the diameter and the center of the pupil using the Hough transform.

After traversing all θ, the point $\left(x_{0},y_{0}\right)$ in the image space is mapped to a parameter space as a circle. It is clear that every edge point in the image space corresponding to the parameter space is a circle, and these circles will intersect at one point. Then, we make statistics on all coordinate points in the parameter space to find the largest accumulated value point which can be supposed as the center. When the radius ranges and edge point coordinates are known, the values of parameters a and b can be found. That is, the center of the image space as well as the corresponding radius will be known. The process of extracting the pupil diameter is shown in Figure 7.

• Fixation duration detection

We use the EOG and video method to record the eye movement information synchronously. Compared with the EOG signals, the video method is not sensitive to some subtle eye movement such as micro-saccades, nystagmus, etc., due to the low sampling ratio. Considering that the fixation is a relative stable state, the detection of fixation mainly focuses on the tendency of signals change rather than details. Thus, we select the video method to perform the fixation detection. In order to reduce differences among different observation signals, we transform the amplitude of raw eye movement signals into the scope of [0, 1], which is named as “normalization”. Specifically, we first compute the absolute values of raw signals, s(t); then, we extract the minimum, *s*_max_, and maximum, *s*_min_, and calculate their difference value; finally, we compute the normalized result, *s*(*t*)_nor_, using the following equation:

(9) $$s{\left(t\right)}_{\mathrm{nor}}=\frac{abs(s(t))-abs(s_{\min})}{abs(s_{\max})-abs(s_{\min})},$$

**Figure 8:**
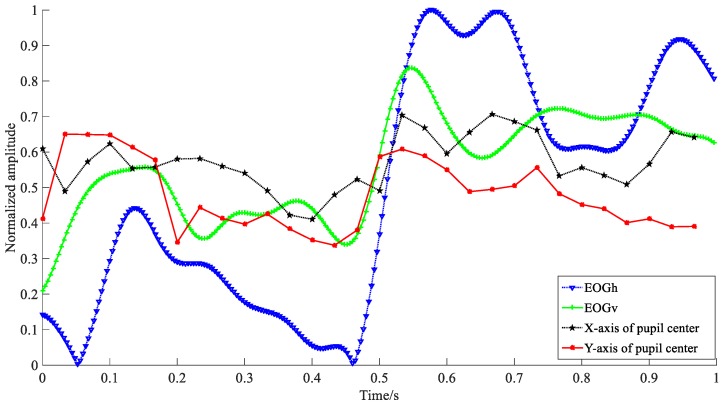
Waveforms of normalized EOG and video signals according to the horizontal and vertical eye movements, receptively. *EOG_h_* and video eye movement in *X*-axis indicate the horizontal eye movement components, *EOG_v_* and *Y*-axis are the vertical eye movement components.

Figure 8 shows the waveforms of normalized EOG and video according to horizontal and vertical directions in 1 s, receptively. Apparently, the amplitude variations of EOG signals are larger than that of video signals. To detect fixation duration, we further compute the change amplitude of *X* and *Y*. Here, the variables of *X* and *Y* are the horizontal and vertical center points of the pupil in the current frame. Their values refer to the index of rows and columns, respectively. The detection procedure can be described as follows:

If the changes of *X* and *Y* are both less than one pixel in the consecutive two frames, i.e., $X_{i+1}-X_{i}\leq 1\;\mathrm{pixel},Y_{i+1}-Y_{i}\leq 1\;\mathrm{pixel}\;(i=1,\cdots ,N)$, it is considered as a possible fixation frame; otherwise, it is the other eye movement frame. Furthermore, to improve the detection accuracy and reduce the confusion of other eye movements (e.g., slow smooth pursuit), we perform the second adjustment for all detected fixation segments by introducing additional detection parameters. That is, if the duration of detected segment is within 100 to 200 ms [28] and $X_{\max}-X_{\min}\leq 3\;\mathrm{pixels},Y_{\max}-Y_{\min}\leq 3\;\mathrm{pixels}$ ($X_{\max}$, $X_{\min}$, $Y_{\max}$, $Y_{\min}$ are the maximums and minimums of center points according to horizontal and vertical directions in a detected possible fixation segment), we determine the current segment is a fixation activity; otherwise, it is not.

### 3.4. Features Fusion for Emotion Recognition

To improve the ability of emotion representation, we further integrate the time-domain and time/frequency-domain features extracted from the eye movement signals. Generally, there are two ways to achieve features fusion: feature level fusion (FLF) and decision level fusion (DLF) [36]. The FLF refers to a direct combination of time/frequency-domain feature vectors and time-domain feature vectors. It therefore has some advantages of low computation load, low complexity, etc. Compared with the FLF, the DLF is a decision-level feature fusion algorithm. Considering the characteristics of eye movement signals, we choose the maximal rule [29] to combine the classification results from each feature classifiers in the present work.

The maximal rule is to compute the maximal values of all the probabilities that a sample belongs to each category in all classifiers and choose the class label with the highest probability. The maximal rule is defined as follows:

(10) $$C(x)=\arg\max\{P_{q}(w_{a}|x)\}{|}_{\begin{array}{l} a=1\cdots k\;\\ q=1\cdots Q \end{array}}$$

where *C* is the output of the decision level fusion based on the maximal rule. *Q* is the ensemble of all classifiers that can be selected for feature fusion, *k* means the total number of all categories. x is testing data, $P_{q}(w_{a}|x)$ is the posterior probability in the category *w_a_* according to the classifier q. To realize the DLF for emotion recognition, we first calculate the posterior probabilities of four features in the case of three emotion categories. Then, the maximum posterior probabilities corresponding to the three categories in features are compared with each other. Finally, we choose the category with the highest posterior probability as the final decision [33].

## 4. Experiments and Results Analysis

**Figure 9:**
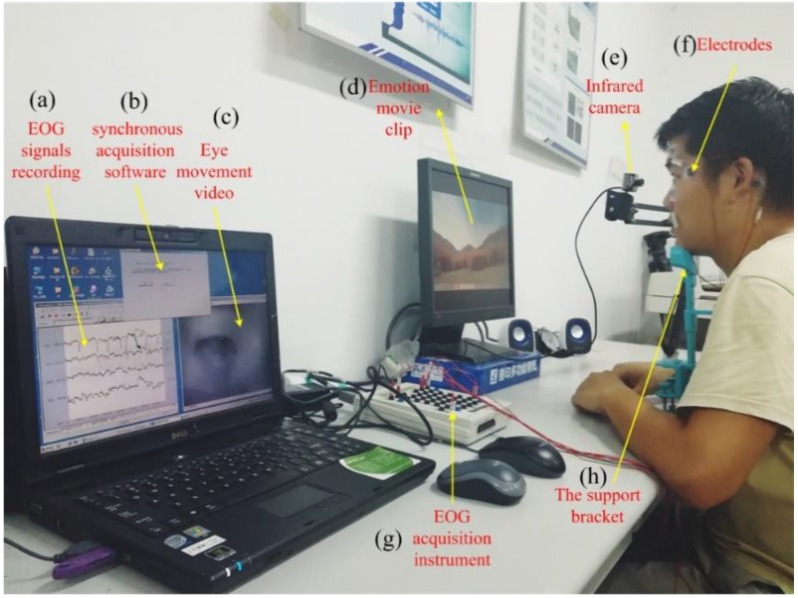
The real experimental scene. (**a**) EOG signals collection and displaying system, (**b**) the synchronous acquisition software, (**c**) the recording eye movement video, (**d**) Emotion movie clips, (**e**) the infrared camera, (**f**) six bio-electrodes used to acquire the EOG signals, (**g**) EOG signals amplifier (Neuroscan), (**h**) the support bracket for holding head.

The experiments have been performed in an illumination-controlled environment. Figure 9 shows a real experimental scene. In our experiment, the total dimension of time/frequency-domain features is 3808 (i.e., each frequency bin has 119 feature parameters and there are 32 frequency bins), and the number of time-domain features is 11. The numbers of all samples collected from each subject are 2160 and 360 samples are used as testing samples.

During the procedure of the experiment, the detection threshold $th_{sac}$ is crucial to determine the accuracy of emotion recognition. In order to obtain the optimum performance, we elaborately choose the 1/3 of the maximum amplitude as the detection threshold by performing the comparison experiments under different threshold values (e.g., 1/4, 1/3, 1/2, and 2/3 of the maximum amplitude). In addition, for the threshold in detecting the pupil diameter, we compute the minimum pixel value (*MPV*) of the grayscale image firstly, and $th_{bi}$ is obtained by using the following equation:

(11) $$th_{bi}=\{\begin{array}{c} 4\times MPV\;(MPV\leq 20)\\ 3\times MPV\;(20<MPV\leq 30)\\ 75\;(MPV>30) \end{array},$$

In the aspect of emotion classification, we adopt the SVM with a polynomial kernel function for both feature and decision level fusion. To calculate the accuracy, we compare the data labels with the output of SVM model. If the predict output is same as the data label, it indicates that the classification is correct; otherwise the classification is incorrect. On this basis, we divide the total number of samples with correct classification by that of all samples. Besides, we also introduce the *F*_1_ score to evaluate the performance of the proposed method. It can be defined as follows:

(12) $$F_{1}\;score=\frac{1}{N}\sum_{i=1}^{N}\frac{2\times precision_{i}\times recall_{i}}{precision_{i}+recall_{i}},$$

where i=1⋯N,N is the category to be classified, precision is the number of correct positive results divided by the number of all positive results returned by the classifier, and the recall is the number of correct positive results divided by the number of positive results that should be returned.

To objectively assess the performance of the proposed algorithm, a 10 × 6 cross-validation method is applied. Specifically, the original labeled database in terms of different eye movement patterns is first randomly partitioned into six equally sized segments: five segments are used to train the SVM model and the rest is employed to test it. Subsequently, 10 repetitions of the above step are performed such that five different segments are held out for learning while the remaining segment is used for testing within each repetition. Finally, the recognition ratios are computed by averaging all results from 10 rounds. In the procedure of cross-validation, the training and testing datasets must cross-over in successive rounds such that each eye movement trial has a chance of being validated against.

### 4.1. Determination of Sliding Window Length

**Figure 10:**
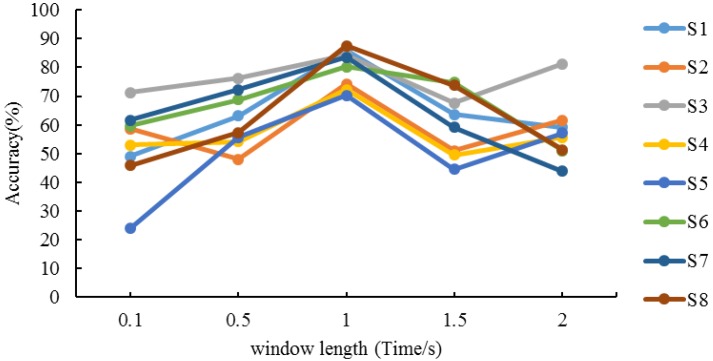
Recognition accuracy of different window lengths.

Because the time resolution of the STFT is determined by the width of the sliding window and the frequency resolution is decided by the spectral width of the window function, the length of sliding window plays an important role on extracting time/frequency-features using STFT. The longer the sliding window width is, the lower the time resolution and the higher the frequency resolution are. Considering the balance between the resolution of the frequency domain and accuracy in different frequency bins, we finally determine the point of STFT is 64. On this basis, we execute the performance evaluation using different length of window, i.e., 0.1 s, 0.5 s, 1 s, 1.5 s, and 2 s. All the number of overlapping sampling points are half the length of the window. The results of different subjects are shown in Figure 10. 

We can see that the recognition ratios for all subjects’ present different performance due to different familiarity degrees to the experimental procedure, data collection instrument, strength of emotion response, cognition requirement, etc. Moreover, the recognition ratios also present difference among different window lengths. On closer inspection of the experimental results within 1–2 s, it turns out that the recognition ratios of S1, S6, S7 and S8 appear a downward trend while S2, S3, S4 and S5 acquire slight increment from 1.5–2 s. On the other hand, the best performance for all subjects are obtained at 1 s, that is, the algorithm achieves the highest accuracy when the length of window is 1 s. Therefore, we determine the optimal length of the sliding window is 1 s.

### 4.2. Performance Evaluation of Emotion Recognition

Experiments have been performed in terms of two steps: the single feature-based recognition and features fusion-based recognition. The former is used to validate the feasibility of using eye movement information to achieve the emotion recognition. The latter is executed to obtain an effective approach for improving the recognition performance. Table 2 shows the recognition results under different single feature conditions. 

As can be seen in Table 2, the average recognition accuracy and *F*_1_ score of time/frequency-domain features achieve 85.37% and 85.65%, respectively, which obtain significant improvement of 30.02% (30.93%), 34.85% (35.43%), and 38.12% (39.08%) compared with features of “saccades”, “fixations”, and “pupil diameters”. The reason for this result is that the proposed STFT-based emotion recognition method not only considers the time-domain change of emotional eye movement, but also analyzes their frequency-domain characteristics. Therefore, the time/frequency features provide more details than the time-domain features for emotion recognition. As for time-domain features, the average accuracies of 55.35%, 50.52%, and 47.25% reveal that they can distinguish different emotions to some extent. Furthermore, we find that subject No. 4 gets the lowest accuracy of 79.81% and 39.44% for time/frequency features and pupil diameters. By analyzing the time-domain waveforms and inquiring the subject, the underlying reason is that stimuli videos cannot induce the emotion successfully due to the difference of cognition. 

**Figure 11:**
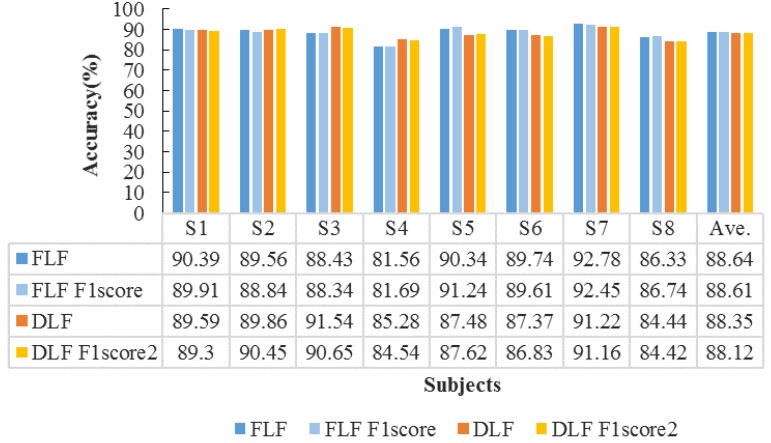
Features fusion-based on classification accuracies (%) for all subjects.

Next, we further perform the features fusion strategies to improve emotion recognition accuracy. The fusion strategies include feature level fusion and decision level fusion with maximal rules (described in Section 3.4). The accuracies of FLF and DLF for all subjects are shown in Figure 11. 

**Figure 12:**
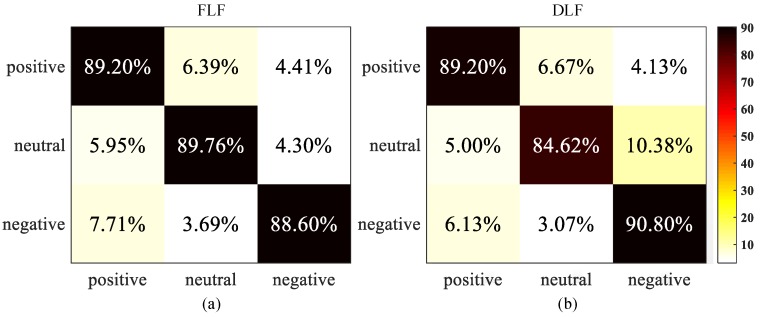
Confusion matrices of two fusion strategies. (**a**) The FLF confusion matrix; (**b**) The DLF confusion matrix. Each row of the matrix represents the actual class and each column indicates the predicted class. The element (i,j) is the percentage of samples in category *i* that is classified as category *j*.

From Figure 11 we can observe that the FLF achieves the average accuracy of 88.64% and average *F*_1_ score of 88.61%; while the DLF with maximal rules obtains the average accuracy of 88.35% and average *F*_1_ score of 88.12%. Since the FLF directly concatenate two feature vectors of EOG and video signals to train the detection model, it can provide more complementary emotion information than DLF. On the other hand, there is still a slight increment of accuracy compared with only using the single feature. Therefore, the recognition performance of the DLF is lower than that of the FLF. To further evaluate their performance, we compute the confusion matrix in the case of three emotions, i.e., positive, neutral, and negative. The results are shown in Figure 12.

In Figure 12, correct recognition results are located at the diagonal and substitution errors are shown on off-diagonal. The largest between-class substitution errors are 7.71% (the FLF method) and 10.38% (the DLF method), respectively. Similarly, the smallest errors are 3.69% (the FLF method) and 3.07% (the DLF method), respectively. Additionally, we can also observe that all emotional states are recognized with very high accuracy over 84%, while there is an obvious confusion among three emotional states. Furthermore, 7.71% and 6.13% negative class are falsely returned positive state in the case of the FLF and the DLF method. These confusions may be caused by personality and preference differences of the adolescents. For example, some teenagers prefer atmospheres in open public areas such as music club or bar, by contrast, others are unwilling to stay such environments. Meanwhile, 6.39% and 6.67% positive class are confused with neutral state. By inquiring the subject’s emotional feeling after experiments, we speculate the discrepancy of adolescents’ perceptual and rational choice might play an important role on this result. Compared the class substitution errors between the FLF and the DLF, most of errors for the DLF is lower than that for the FLF, the average accuracy of the FLF is higher than the DLF. This experimental result reveals that the FLF strategy can effectively decrease the confusion among different emotional states. Generally, the FLF and the DLF obtain an obvious improvement of 29.11% and 28.82% compared with the accuracy of single feature-based method, which proves that the effectiveness of the features fusion-based method.

## 5. Conclusions

This study presented an emotion recognition method combining EOG and eye movement video. To improve the performance of emotion perception, we further explored two fusion strategies (i.e., the FLF and the DLF) to integrate the time/frequency features, saccades features, fixation features, and pupil diameters. Experimental results proved that the proposed eye movement information-based method can effectively distinguish three emotions (positive, neutral, and negative), which provides a promising approach to monitor the emotion health.

Since the eye movements can reflect the emotional state, as an effective supplement to EEG emotion recognition, the proposed method can not only be applied to E-healthcare applications such as monitoring, diagnosing, and preventing mental disorders, but also be employed in the emotion safety evaluation for the people engaged in high-risk work (e.g., drivers, pilots, soldiers, etc.). Besides, it provides an alternative communication way for the patients who are suffering from motor diseases such as Amyotrophic Lateral Sclerosis (ALS), Motor Neuron Disease (MND), injured vertebrae, etc., while still retaining coordination of brain and eye movements.

Because the participants are asked to maintain the stability of their heads as much as possible, the practicability of the proposed method will decrease. Additionally, additional eye movement components such as dispersion, micro-saccades, slow-speed eye movement, etc., were not involved in the present work due to the difficulties in acquiring them. To address these problems, we will prioritize three aspects of research in the further work: (1) use mean shift tracking algorithm with adaptive block color histogram to develop a robust pupil detection algorithm; (2) improve the data collection diagram and analysis algorithm to obtain more eye movement components. Independent component analysis (ICA), as an effective method for the blind source separation, might be a good choice to acquire new emotion-related eye movement components; (3) expand the scale of training and testing dataset in order to further prove the effectiveness of the proposed algorithm.

## Figures and Tables

**Table 1:** Time-domain Eye Movement Features.

Parameters	Time-Domain Features
Saccade	the maximum, mean, standard deviation of saccade duration, and saccade frequency
Fixation	the maximum, mean, standard deviation of fixation duration, and fixation frequency
Pupil diameter	the maximum, mean, standard deviation

**Table 2:** Classification accuracies (%) and average *F*_1_ score (%) under single feature conditions.

Subjects	Time-Frequency		Saccades		Fixations		Pupil Diameters		Ave.	
	Accuracy	*F*_1_ Score	Accuracy	*F*_1_ Score	Accuracy	*F*_1_ Score	Accuracy	*F*_1_ Score	Accuracy	*F*_1_ Score
S1 (f) ^1^	87.69	88.78	52.64	51.83	55.94	56.23	42.7	43.45	59.74	60.07
S2 (f)	87.6	87.1	60.4	58.93	49.82	47.56	51.94	52.17	62.39	61.44
S3 (m)	89.51	89.6	53.12	52.63	44.07	44.78	55.21	54.73	60.48	60.44
S4 (f)	79.81	81.21	54.89	55.54	49.43	48.79	39.44	40.05	55.89	56.4
S5 (f)	82.22	83.67	54.72	54.89	48.27	47.8	49	48.78	58.55	58.79
S6 (f)	83.61	83.5	58.89	60.13	49.63	48.59	45.17	46.89	59.32	59.78
S7 (m)	88.28	88.1	50.27	48.36	60.94	62.87	45.39	43.1	61.22	60.61
S8 (m)	83.27	83.22	57.89	55.46	46.22	45.14	49.11	43.42	58.62	56.81
Ave.	85.37	85.65	55.35	54.72	50.52	50.22	47.25	46.57	59.53	59.62

^1^ S1–S8 are indexes of subjects, the symbols “f” and “m” means female and male, respectively.
